# Dual degradation signals destruct GLI1: AMPK inhibits GLI1 through β-TrCP-mediated proteasome degradation

**DOI:** 10.18632/oncotarget.17769

**Published:** 2017-05-10

**Authors:** Rui Zhang, Sherri Y. Huang, Kay Ka-Wai Li, Yen-Hsing Li, Wei-Hsuan Hsu, Guang Jun Zhang, Chun-Ju Chang, Jer-Yen Yang

**Affiliations:** ^1^ Department of Basic Medical Sciences, West Lafayette, Indiana, USA; ^2^ 5/F of Cancer Centre, Prince of Wales Hospital, Department of Anatomical and Cellular Pathology, The Chinese University of Hong Kong, Shatin, Hong Kong; ^3^ Center for Cancer Research, Purdue University, West Lafayette, Indiana, USA; ^4^ Department of Comparative Pathobiology, Purdue University, West Lafayette, Indiana, USA

**Keywords:** AMPK, β-transducin repeat containing protein (β-TrCP), Hedgehog, GLI1, medulloblastoma

## Abstract

Overexpression of the GLI1 gene has frequently been found in various cancer types, particularly in brain tumors, in which aberrant GLI1 induction promotes cancer cell growth. Therefore, identifying the molecular players controlling GLI1 expression is of clinical importance. Previously, we reported that AMPK directly phosphorylated and destabilized GLI1, resulting in the suppression of the Hedgehog signaling pathway. The current study not only demonstrates that AMPK inhibits GLI1 nuclear localization, but further reveals that β-TrCP plays an essential role in AMPK-induced GLI1 degradation. We found that activation of AMPK promotes the interaction between β-TrCP and GLI1, and induces β-TrCP-mediated GLI1-ubiquitination and degradation. Inhibiting AMPK activity results in the dissociation of the β-TrCP and GLI1 interaction, and diminishes β-TrCP-mediated-GLI1 ubiquitination and degradation. On GLI1, substitution of AMPK phosphorylation sites to aspartic acid (GLI1^3E^) results in stronger binding affinity of GLI1 with β-TrCP, accompanied by enhanced GLI1 ubiquitination and later degradation. In contrast, the GLI1 alanine mutant (GLI1^3A^) shows weaker binding with β-TrCP, which is accompanied by reduced β-TrCP-mediated ubiquitination and degradation. Together, these results demonstrate that AMPK regulates GLI1 interaction with β-TrCP by phosphorylating GLI1 and thus both post-translational modifications by AMPK and β-TrCP ultimately impact GLI1 degradation.

## INTRODUCTION

The Hedgehog (Hh) pathway is an essential regulator of the differentiation, development and proliferation of cells, organs and tissues [1, 2]. Aberrant activation of Hh pathway is oncogenic in animal models and human patients [1, 3]. In human, mutations in Hh major components (PTCH1, SMO) are associated with sporadic and familial skin cancers (basal cell carcinoma, BCC), brain tumors (medulloblastoma, MB) and rhabdomyosarcoma (RMS) [3, 4].

Hh signaling controls transcription of target genes by regulating the activities of three Glioma-associated oncogenes (GLI1-3) transcription factors. The GLI proteins are zinc finger transcription factors with five sequential zinc fingers of the C2H2 type of DNA binding domain [5]. GLI1 is the first found Hh relative gene amplified in a human glioma [6]. Overexpression of GLI1 protein has been described in various tumor types such as MB [7], BCC [8], RMS [9, 10], prostate [11, 12], biliary [13], breast [14–16], lung [17], colon [18, 19] and bladder [20] cancers. Moreover, GLI1 expression level is highly correlated with disease status and patient survival. For example, higher GLI1 expression is associated with more advanced (and metastatic) tumors [11, 13, 16] and low expression of GLI1 confers longer survival in patients with oral squamous cell carcinoma (SCC) [21]. This indicates that GLI1 could serve as a prognosis and diagnosis marker for cancer patients.

GLI1 not only functions as a Hh transcription activator but also a target gene of Hh signaling, and expression of GLI1 mRNA and protein is considered a measure of Hh activity [1, 22]. Higher GLI1 expression can be the result of either ligand-dependent or -independent intrinsic Hh pathway activation [23]. For example, mutations at PTCH1, SMO and SUFU have been found in BCC and MB, and result in an increased expression of GLI1 [24, 25]. In addition to canonical regulatory steps, post-translational modifications of GLI proteins alter Hh /GLI activity [3]. For example, GLI1 is activated by oncogenic kinases such as AKT [26], MAPK/ERK [27], KRAS [28], mTOR/S6K1 [29] and aPKC [30] through a non-canonical regulation. These oncogenic kinases promote GLI1 transcriptional activity through different mechanisms. For example, atypical Protein Kinase C ι/λ (aPKC) has been identified as both a GLI1 target and a regulator of GLI1 activity in BCC. Phosphorylation of GLI1 by aPKC displays enhanced DNA binding and transcriptional activity in BCC [30]. Conversely, several kinases such as PKA [31] and PKCδ [32] serve as negative regulators of GLI1. Recently, AMPK was found to phosphorylate GLI1 and destabilize GLI1 protein levels, ultimately leading to inhibition of the Hh pathway [33, 34]. However, the underlying mechanism of how AMPK induces GLI1 degradation still remains unclear.

The *Drosophila* homolog of GLI, cupitus interruptus (Ci), is regulated by ubiquitin E3 ligases and kinases in response to Hh signaling. Hh activation blocks proteolytic processing of full-length Ci to generate a truncated repressor form. Jiang et al. reported that casein kinase I (CKI) family members phosphorylate Ci and that phosphorylation on Ci confers binding to β-TrCP, an E3 ligase, followed by proteasome-meditated proteolytic processing to generate a repressor form of Ci [35]. β-TrCP, itself demonstrated to be extremely important for tumorigenesis, is known to mediate the ubiquitination and degradation of GLI1-3, the vertebrate homologs of Ci [8, 36–38]. But how upstream signals or kinase modifiers control β-TrCP-mediated GLI1 degradation remains to be elucidated.

We show that overexpression of GLI1 promotes MB and fibroblast cell lines proliferation and colony formation in a soft agar assay. In addition, there is an inverse correlation between phosphor-AMPK and GLI1 protein expression levels in a cohort of brain tumor tissues. Mechanistically, AMPK activation induces GLI1 cytosolic localization, while inhibition of AMPK enhances GLI1 nuclear localization. Furthermore, we identify β-TrCP as a critical mediator for AMPK-induced GLI1 destabilization. AMPK promotes the interaction between β-TrCP and GLI1, as well as β-TrCP-mediated ubiquitination and degradation. In the end, our results provide strong evidence of how AMPK inhibits GLI1 through β-TrCP-mediated ubiquitination and degradation and demonstrate post-translational modifications from two different enzymes coordinately control Hh transcription activator GLI1 which is critical for regulation of cell growth.

## RESULTS

### Overexpression of GLI1 in cancers promotes cell growth

**Figure 1:**
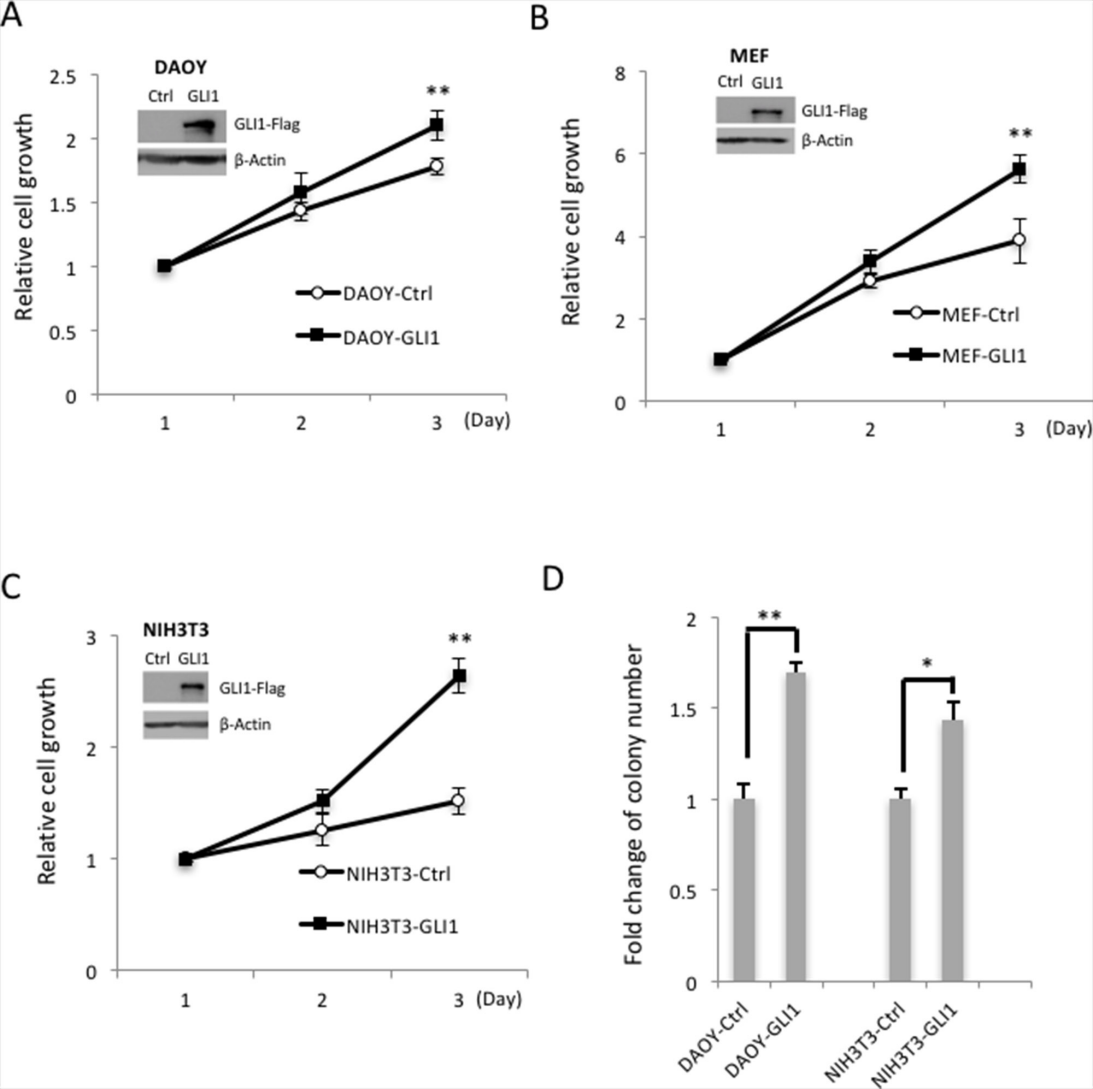
GLI1 promotes MB cell growth and colony formation **(A)** GLI1 protein levels were determined by Western blotting in GLI1 stably overexpressed Daoy-GLI1 (A), MEF-GLI1 **(B)** and NIH3T3-GLI1 **(C)** by comparing with corresponding control cells (Inserted panel). The cell growth assay was examined by MTT assay. The line charts show the means of three independent experiments, and error bars show standard deviations. **(D)** The GLI1 function on anchorage- independent growth was determined by soft agar assay. The cells (DAOY-Ctrl/GLI1 and NIH3T3-Ctrl/GLI1) were seeded on agarose plates for colony formation assays, and colonies larger than 0.2 mm were counted two weeks later. The histogram shows the relative fold changes of three independent experiments, and error bars show standard deviations. (“*” *p* < 0.05, “**” *p* < 0.01)

To examine *GLI1* gene expression across different cancer types, we searched the cBioPortal, the most comprehensive Cancer Genomics database, from Memorial Sloan-Kettering Cancer Center. We found that the *GLI1* gene is highly amplified, mutated and deleted in various cancer types, and that the majority of *GLI1* gene amplification was detected in ∼10% glioblastoma tumor patients (Supplementary Figure 1A). We also searched the Gene Expression Omnibus (GEO) from NCBI database, and found that in glioma, higher GLI1 expression is significantly correlated with higher tumor grades (Supplementary Figure 1B). To examine GLI1 function, we generated three GLI1-stable expression cell lines in a human medulloblastoma cell line (DAOY) and two mouse cell lines (NIH3T3 and mouse embryonic fibroblast cells, MEF). We found that overexpression of GLI1 promotes cell growth in all of the three GLI1 stable cell lines and soft agar colony formation in DAOY and NIH3T3 GLI1 stable cell lines (Figure 1A–1D). These data show that GLI1 plays an essential role in controlling cell growth, and that aberrant overexpression of GLI1 will lead to uncontrolled cell growth in cancer and brain tumor malignance. Thus, identification of the negative GLI1 regulators to control GLI1 protein level and activity is critical for inhibition of cancer cell growth.

### AMPK blocks GLI1 nuclear translocation

**Figure 2:**
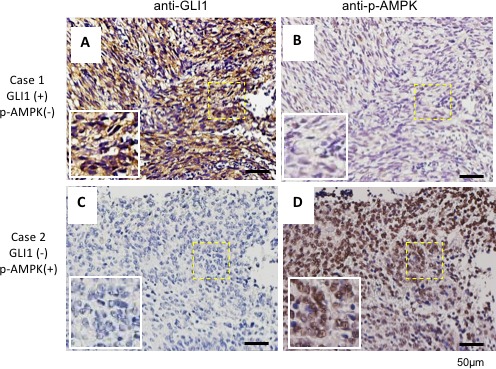
GLI1 expression inversely correlates with p-AMPK in human brain cancer tissues A cohort of 198 human brain cancer samples which included 35 medulloblastoma samples tiled on a tissue array were analyzed by immunohistochemical staining with an anti-GLI1 antibody **(A and C)** and an anti-p-AMPK antibody **(B and D)**. Representative images were consecutive sections of two different MB patients. Scale bar: 50 μm

Previously, we found that AMPK directly destabilized GLI1 and that activation of AMPK inhibits cell growth [33]. Currently, in a cohort of 198 brain tumors, we found that p-AMPK (the active form of AMPK) protein expression levels are inversely correlated with GLI1 as shown in the representative MB cases (Figure 2A–2D and Table 1). Furthermore, AMPK binds with GLI1 (Supplementary Figure 2A), and the binding affinity of AMPK and GLI1 increased ∼9 fold under treatment with AMPK activator 2-deoxylglucose (2DG), in the presence of proteasome inhibitor MG-132 (10 μM) for 6 hours prior to harvest (Supplementary Figure 2B). These results support our previous finding that AMPK serves as a negative regulator of Hh/GLI1 signaling.

**Table 1:** Inverse correlation between GLI1 and p-AMPK expression in human brain cancers

Count		p-AMPK		Total
		**+**	**−**	
**Gli1**	**+**	6	23	29
	−	79	90	169
Total		85	113	198

Odds ratio=0.2 (95% CI 0.1-0.8; p=0.009)

A cohort of 198 human brain cancers tiled in a tissue array which included 35 medulloblastoma samples were analyzed by immunohistochemical staining using antibodies specific to GLI1 and p-AMPK antibody. The association between the expression pattern of GLI1 and p-AMPK was assessed by Pearson's chi-square test and odds ratio (OR). The 95% confidence interval (CI) of the OR is reported. Note that an OR <1 indicates a negative association.

**Figure 3:**
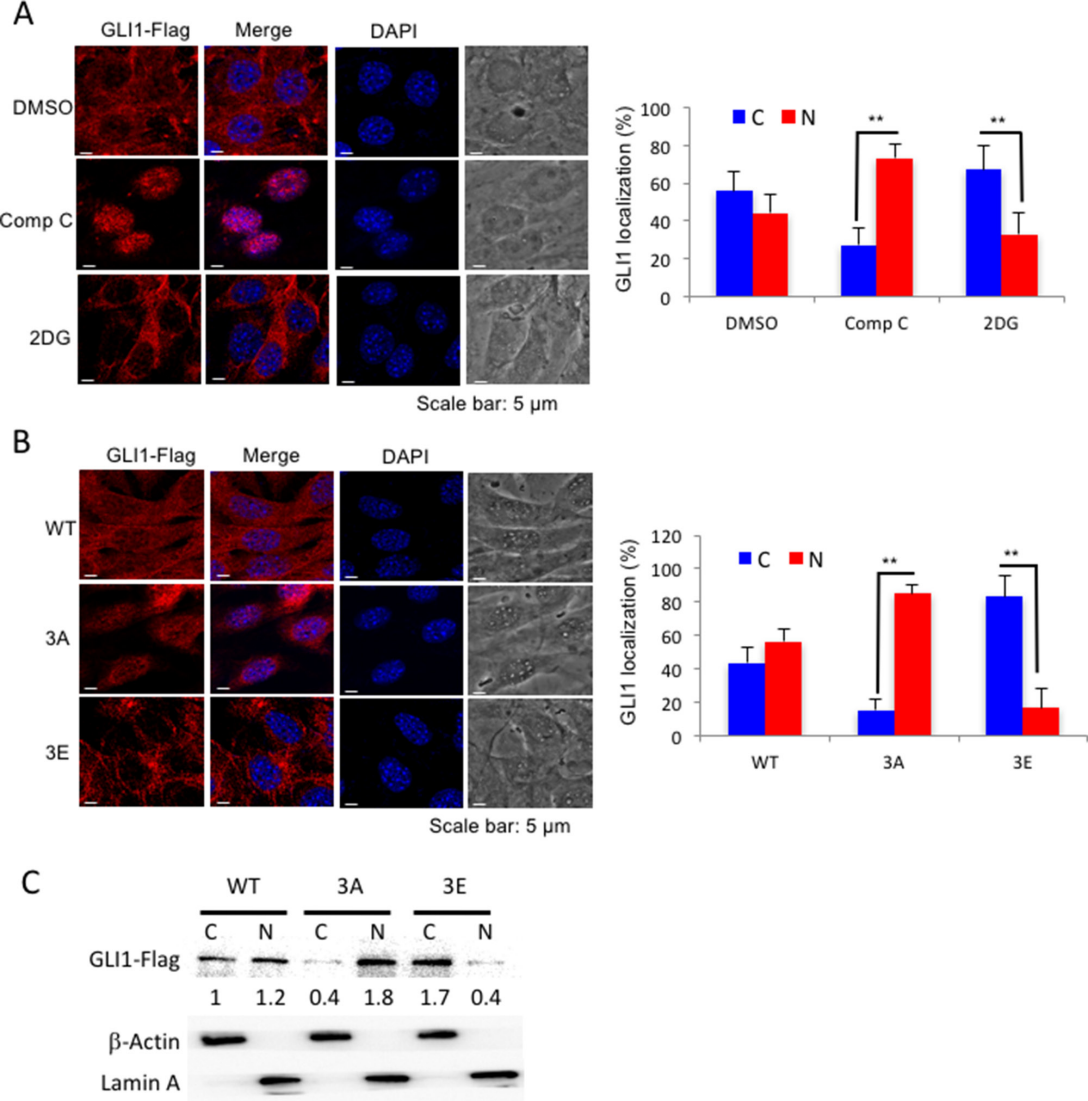
AMPK controls GLI1 protein localization **(A)** NIH3T3 GLI1-Flag stable cells were treated with Comp C (10 μM) for 6 hours and 2DG (25 mM) for 30 minutes, and cells were analyzed by immunofluorescence staining with anti-Flag antibody and DAPI. Bar graphs showed the percentages of cells with cytosolic (C) or nuclear (N) GLI1 expressions. **(B)** NIH3T3 GLI1-flag stable cells (GLI1^WT^, GLI1^3A^ and GLI1^3E^) were treated with MG-132 (10 μM) for 4 hours before cells were analyzed by immunofluorescence staining with anti-Flag antibody. All the results were from three independent immunofluorescence experiments and error bars show standard deviations. (“**” *p* < 0.001) **(C)** Cell lysates from (B) were subjected to nuclear and cytoplasmic fractionation and analyzed by immunoblotting with indicated antibody. The numbers showed the intensity of GLI1-Flag bands analyzed by Image J and normalized with cell fractionation loading controls and compared with GLI1^WT^ cytosolic level. Cytosolic marker (C): β-Actin; Nuclear marker (N): Lamin A.

Since GLI1 is an Hh pathway transcriptional activator, we asked whether phosphorylation of GLI1 by AMPK prevents GLI1 translocation to the nucleus and promotes retention of GLI1 in the cytoplasm. First, we treated the GLI1-flag stable MEF cell line with either an AMPK inhibitor (Compound C, Comp C) or AMPK activator (2DG) and examined GLI1 localization by immunofluorescence staining. In Comp C-treated cells, GLI1-flag protein mainly localized to the nucleus; however, in the 2DG 30 min treatment group, where a short treatment time enables the GLI1 protein to be detected before degradation, most of the GLI1-flag protein was retained in the cytosol (Figure 3A). Three sites of AMPK phosphorylation on GLI1 were previously identified: serine 102, 408 and threonine 1074 [33]. Mutation of all three of these AMPK phosphorylation sites to alanine (GLI1^3A^) prevents AMPK phosphorylation and enhances cell growth but not in GLI1^3E^ (Glutamic acid mutations of GLI1). Similarly, in GLI1^3A^ cells as like in the Comp C-treated cells, GLI1 protein was mainly localized in the nucleus. In GLI1^3E^cells as like the 2DG-treated cells, most of GLI1 protein was localized in the cytoplasm (Figure 3B). Furthermore, those results were consistent with the nuclear and cytoplasm fractionation assay, GLI1^3A^ protein was mainly detected in the nuclear fraction (N) in comparison with the cytosolic fraction (C), while most of GLI1^3E^ was detected in the cytosolic fraction (C) in the immunoblotting experiment (Figure 3C). Together, these results demonstrate that AMPK phosphorylation of GLI1 inhibits GLI1 nuclear translocation and that inhibition of AMPK activity enhances GLI1 nuclear localization.

### β-TrCP is required for AMPK induced-GLI1 degradation

**Figure 4:**
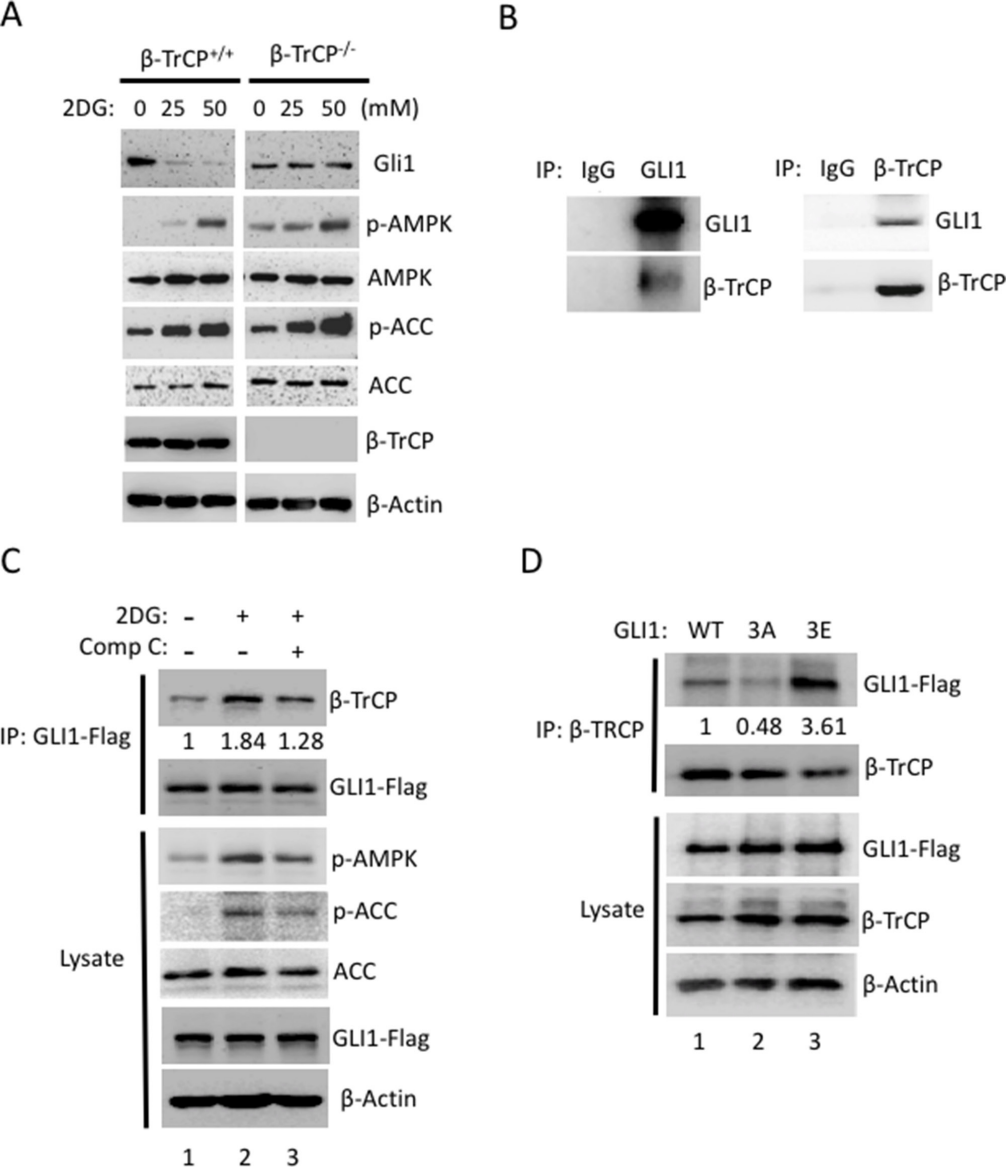
β-TrCP is essential for AMPK-mediated Gli1 degradation **(A)** Both *β-TrCP* wild-type (^+/+^) and *β-TrCP* knockout (^−/−^) MEFs were treated with 2DG (0, 25, 50 mM) for 4 hours. Lysates were collected and analyzed by Western blot using the indicated antibody. **(B)** Reciprocal co-IP assays were performed with antibodies against GLI1 or β-TrCP to pull down GLI1 or β-TrCP protein in pZp53Med1 (Med1) cells. Western blots were performed with GLI1 and β-TrCP antibodies in both IP samples. **(C)** Flag-GLI1 was transiently transfected into HEK293 cells for 48 h and followed by treatment with 2DG (25 mM) with or without Comp C (20 μM) for 30 mins. Immunoblotting for Flag-GLI1 or β-TrCP was performed following IP of Flag-GLI1. WB from the lysate indicates that the Flag-GLI1 transfection and the compound treatments proceeded as expected as p-AMPK and p-ACC levels are increased by 2DG (lane 2) but decreased by Comp C (lane 3). The WB intensity was measured by Image J and the annotated number indicates the ratio of β-TrCP to GLI1 levels normalized to control group. **(D)** Immunoblot for either Flag-GLI1 or β-TrCP using lysates from the IP of β-TrCP from GLI1^wt^, GLI1^3A^ and GLI1^3E^ stably expressed NIH3T3 cells with MG-132 (10 μM) for 4 hours. The WB intensity was measured by Image J and the annotated number indicates the ratio of GLI1 to β-TrCP levels normalized to Gli1^WT^ group.

We reported that activation of AMPK suppressed GLI1 transcriptional activity and led to Hh inhibition [33], but how does AMPK regulate GLI1 protein stability and inhibit its protein level? It was known that E3 ligase β-TrCP degraded GLI1 [8, 39] and therefore we speculated that β-TrCP is involved in AMPK-induced GLI1 degradation. To answer this question, β-TrCP wild-type and knockout MEF cells were treated with or without 2DG and the lysates were analyzed by Western blot (WB). As expected, GLI1 protein was reduced in the 2DG-treated *β-TrCP^+/+^* cells and both p-AMPK and p-ACC (AMPK substrate) levels were increased (Figure 4A). It is important to note that in the *β-TrCP^−/−^* MEFs, no sign of GLI1 protein degradation was detected even under higher concentrations of 2DG treatment (Figure 4A). This result indicates that β-TrCP serves an essential role in AMPK-induced GLI1 degradation.

Post-translational phosphorylation increases the functional diversity of the proteome through impacting conformational changes and protein-protein interactions. To better understand the role of β-TrCP in mediating AMPK-induced GLI1 degradation, we examined the GLI1 and β-TrCP protein-protein interaction in response to AMPK activity. GLI1 was known to interact with β-TrCP in an artificial overexpression system in NIH3T3 cells [8]. We showed that endogenous GLI1 interacted with β-TrCP reciprocally in mouse MB cell line, Med1 cells (Figure 4B). Furthermore, we modulated AMPK activity using an AMPK activator (2DG) or inhibitor (Comp C) in GLI1-transfected HEK293 cells and assessed the GLI1 and β-TrCP interaction with immunoprecipitation/ Western blot (IP/WB). Under activation of AMPK by 2DG, the binding affinity of GLI1 to β-TrCP increased 1.84 fold when compared with the non-treated group (Figure 4C, lane 2 vs lane 1). This interaction was subsequently reduced from 1.84 to 1.28 fold with addition of Comp C (Figure 4C, lane 2 vs lane 3), and lysates showed that p-ACC and p-AMPK levels were increased in the presence of 2DG and decreased in cells co-treated with 2DG and Comp C. The proteasome inhibitor (MG-132) was used to prevent GLI1 degradation in all groups. In a parallel experiment to examine the binding affinity of GLI1 mutants and β-TrCP, we transfected HEK293 cells with GLI1^WT^, GLI1^3A^ and GLI1^3E^ expression vectors and immunoprecipitated β-TrCP and immunoblotted with flag antibody. We found that the binding affinity of GLI1^3A^ and β-TrCP was reduced 52% in comparison with the binding of GLI1^WT^ and β-TrCP (Figure 4D, lane 2 vs lane 1). In contrast, the binding affinity of GLI1^3E^ and β-TrCP was increased 3.61 fold in comparison with the binding of GLI1^WT^ and β-TrCP (Figure 4D, lane 3 vs lane 1). Together, these results demonstrate that AMPK activation induces GLI1 phosphorylation and increases the binding affinity of GLI1 and β-TrCP. Inhibition of AMPK activity, on the other hand, reduces GLI1 phosphorylation and decreases the binding affinity of GLI1 and β-TrCP.

### Activation of AMPK promotes β-TrCP-mediated GLI1 ubiquitination

**Figure 5:**
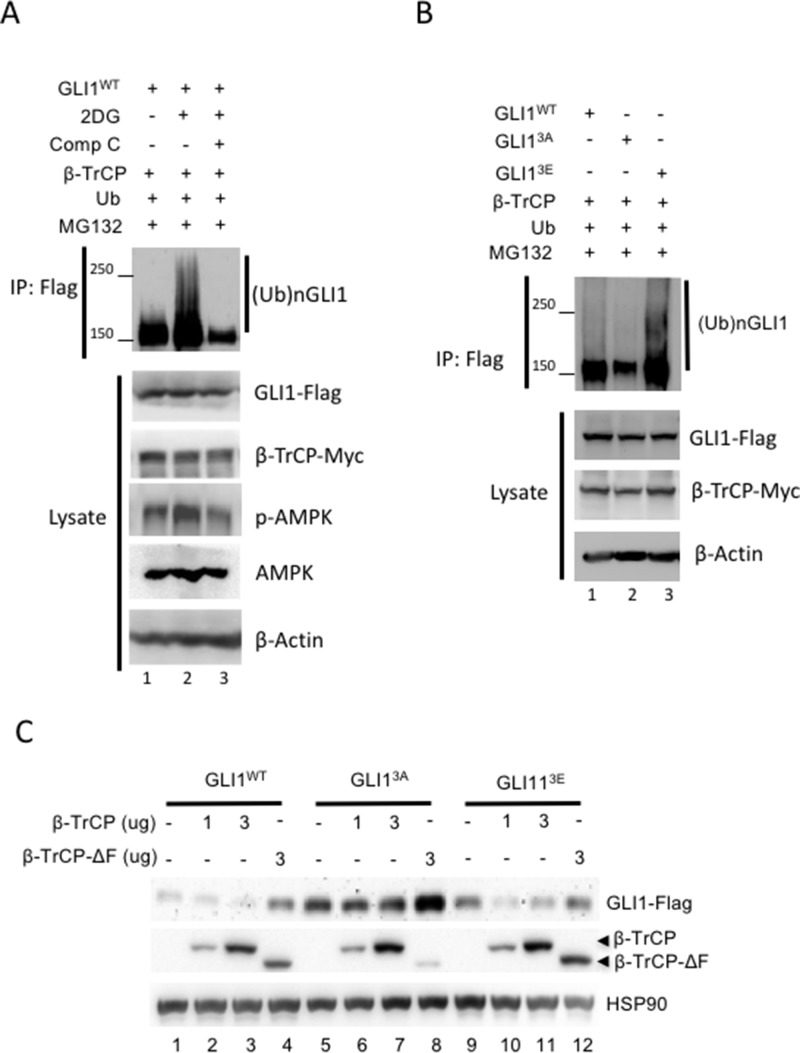
AMPK promotes β-TrCP-mediated GLI1 ubiquitination and degradation **(A)** HEK293 cells were co-transfected with β-TrCP, Ub and Flag-tagged GLI1^wt^ for 36 hours and then treated with MG-132 (10 μM) for 6 hours prior to harvest. The lysates were immunoprecipitated and immunoblotted with anti-Flag antibody. Smear bands show GLI1 ubiquitination. **(B)** HEK293 cells were co-transfected with β-TrCP, Ub and Flag-tagged GLI1^wt^, GLI1^3A^ and GLI1^3E^ for 36 hours and treated with MG-132 (10 μM) for 6 hours prior to harvest. Ubiquitination of GLI1 was analyzed from IP of GLI1-Flag in WB with anti-Flag antibody. **(C)** Flag-tagged GLI1^wt^, GLI1^3A^ or GLI1^3E^ was co-transfected with Myc-tagged β-TrCP (1 and 3 μg DNA) and β-TrCP ΔF (3 μg) for 36 hours in HEK293 cells. Lysates were harvested for immunoblotting with indicated antibody.

To further examine whether activation of AMPK promotes β-TrCP mediated-GLI1 ubiquitination, an *in vivo* ubiquitination assay was performed. First, we confirmed that GLI1 was ubiquitinated by β-TrCP (Supplementary Figure 3, lane 2 vs 4). Deletion of the β-TrCP F-box domain resulted in significant reduction of GLI1 ubiquitination; the β-TrCP-ΔF mutant is most likely still able to bind to GLI1 [40], but unable to initiate ubiquitin modification with loss of its Skp1 interaction domain. Next, GLI1, β-TrCP, and ubiquitin (Ub) expression vectors were transfected in HEK293 cells which were then treated with 2DG or 2DG plus Comp C prior to lysate harvest and IP with flag antibody and analysis by WB. The results showed that GLI1 was highly ubiquitinated in cells treated with 2DG (Figure 5A, lane 2 vs lane 1), but ubiquitination was completely abolished in cells treated with both 2DG and Comp C (Figure 5A, lane 3 vs lane 2). To further study whether enhanced GLI1 ubiquitination under AMPK activation is due to the phosphorylation of GLI1 protein by AMPK, we transfected GLI1^WT^, GLI1^3A^, GLI1^3E^, β-TrCP and Ub plasmid DNAs into HEK293 cells. Lysates were collected and IPs were performed with flag antibody followed by analysis by WB. GLI1^3E^exhibits a strong ubiquitination pattern (Figure 5B, lane 3) but GLI1^3A^ubiquitination was dramatically reduced (Figure 5B, lane 2). Together, these results show that activation of AMPK not only promotes the GLI1/ β-TrCP interaction but also increases β-TrCP-mediated GLI1 ubiquitination.

### AMPK activation enhances β-TrCP-mediated GLI1 degradation

To further examine whether AMPK promotes β-TrCP-mediated GLI1 degradation, we transfected GLI1^WT^, GLI1^3A^, GLI1^3E^ individually with β-TrCP or β-TrCP-ΔF into HEK293 cells for 36 hours and collected the lysates. Samples were analyzed by WB. Results showed that both GLI1^WT^ and GLI1^3E^ proteins were decreased with β-TrCP transfection (Figure 5C, lane 3 vs 1 and lane 11 vs 9); however, GLI1^3A^ protein remained at similar levels even under higher β-TrCP expression (Figure 5C, lane 7 vs 5). As expected, β-TrCP-ΔF had no effect on the reduction of GLI1 protein levels in all GLI1 groups (Figure 5C, lane 4, 8, 12). However, β-TrCP-ΔF stabilized GLI1 protein in the GLI1^WT^, GLI1^3A^ and GLI1^3E^ group (Figure 5C, lane 4 vs 1, lane 8 vs 5, and lane 12 vs 9), which could result from the competing binding to GLI1 between exogenous β-TrCP-ΔF and endogenous GLI1 degradation factors such as endogenous β-TrCP. We also measured GLI1 protein stability in β-TrCP knockout cells. In cells treated with both 2DG and cycloheximide (CHX), the abundance of GLI1 protein was decreased in a time-dependent manner in *β-TrCP*^+/+^ cells but not in *β-TrCP*^−/−^ cells (Supplementary Figure 4). Together, these data demonstrate that AMPK inhibits GLI1 protein levels through β-TrCP-mediated ubiquitination and degradation.

## DISCUSSION

Hh signaling has been demonstrated to be a key pathway in tumor initiation and progression [41]. Several studies have demonstrated the prognostic value of Hh signaling proteins in cancer patients [12, 16, 18]. Cancer signaling transduction can recapitulate dysregulated developmental signaling, so understanding mechanisms of Hh/Gli1 pathway regulation is critical to developing therapeutic targets for the cancer therapy.

Recently, two groups including ours have reported that AMPK directly phosphorylates GLI1 and that activation of AMPK inhibited GLI1 protein levels and Hh signaling [33, 42]. AMPK phosphorylation-resistant GLI1^3A^ mutant demonstrates increased protein stability [33]. Blockage of new protein translation with cycloheximide demonstrated that the increased GLI1 protein level was most likely due to an effect on post-translational stability. However, the mechanism for this post-translational stability was unknown. In other studies, β-TrCP had been identified as an E3 ligase that degrades Gli1 [8, 39]. GLI1 is a transcriptional activator that translocates to the nucleus, and the dynamics of how GLI1 interaction with β-TrCP is not fully identified. In current study, we reveal a novel, specific regulation in the β-TrCP/GLI1 dynamic by demonstrating that AMPK phosphorylation of GLI1 is important for β-TrCP-mediated degradation. In addition, AMPK phosphorylation on GLI1 induced GLI1 cytoplasmic localization, enhanced GLI1 binding affinity with β-TrCP and promoted β-TrCP-mediated GLI1 ubiquitination and degradation.

Post-translational modifications are important for regulation of protein-protein interactions, protein stability and localization [43, 44]. GLI1 that is resistant to AMPK phosphorylation on three of its residues (serine 102 and 408 and threonine 1074) demonstrated prolonged protein stability [33]. In this study, our results showed that activation of AMPK decreased translocation of GLI1 to the nucleus and that this was dependent on AMPK phosphorylation of GLI1 at its three phosphorylation sites. Nuclear translocation is important for the role of GLI1 as a transcriptional activator. Finally, we showed that the phosphorylation marks on GLI1 are important for its binding to β-TrCP and ultimately its ubiquitination and degradation. Modulating the phosphorylation state of GLI1 therefore impacts its protein stability and activity and has a significant therapeutic potential.

AMPK phosphorylation-resistant GLI1^3A^ protein localized predominantly to the nucleus compared to GLI1^3E^ which was detected predominantly in the cytoplasm. This suggests that AMPK phosphorylation on the three GLI1 phosphorylation sites is important for GLI1 nuclear or cytoplasmic compartmentalization. In addition, the finding that GLI1^3A^is predominantly nuclearly localized is consistent with our reports that, compared with GLI1^wt^ and GLI1^3E^, the GLI1^3A^ mutant shows an increased oncogenic potency (29). We demonstrated previously that AMPK activation reduced GLI1 transcriptional output as measured by mRNA levels of *Ptch1* and *Gli1*, both target genes of GLI1 [33]. It is possible that this reduction in GLI1 transcriptional output was due in part to the relative decrease of GLI1 protein in the nucleus versus in the cytoplasm. However, the role of the three AMPK phosphorylation sites on GLI1 in mediating GLI1/DNA binding is yet unexamined. The binding of GLI1 to the promoters of its target genes impacts the efficacy of GLI1 activity, so whether AMPK activation and phosphorylation of GLI1 on its target residues affects GLI1 promoter binding should be examined in a future study.

In the case of multi-site post-translational modifications, the activity of a protein can depend on the specific combination of modifications. For example, protein kinase A (PKA) has been shown to phosphorylate GLI2/3 on six residues, with phosphorylation on the first four residues resulting in degradation of the transcription factors and phosphorylation on all six blocks resulting in the conversion of GLI2/3 into transcriptional activators [45]. Previously we have shown that mutating GLI1 on all three AMPK phosphorylation sites to alanines was sufficient to increase oncogenic potency of cells [33]. In this study, we demonstrated that resistance of GLI1 to AMPK phosphorylation of these three residues resulted in GLI1 nuclear localization and reduced binding to β-TrCP. How the combination of these three residues interacts or interferes with modifications from other kinases remains to be examined such as PKA and S6 kinase I (S6K1). PKA phosphorylation on GLI1 Thr-374 results in retention of GLI1 in the cytoplasm and decrease of transcriptional activity, while its phosphorylation of GLI1 on Ser-640 is also inhibitory of GLI1 function [46]. Another kinase, ribosomal protein S6K1, downstream of mTOR signaling and known to be inhibited by AMPK, activates GLI1 activity by phosphorylating it on Ser-84 [29]. Whether Ser-84 phosphorylation impedes AMPK-mediated phosphorylation or Ser-102, Ser-408 and Thr-1074 prohibits S6K1-mediated phosphorylation remains to be determined.

Together, the “phosphorylation code” created by the phosphor-marks from different kinases controls GLI1 activity, similar to how different combinations of modifications by PKA impact GLI2/3 activation or degradation. This is consistent with our findings that AMPK phosphorylation regulates the affinity of GLI1 to β-TrCP and its subsequent ubiquitination. Interestingly, AMPK regulatory sites on GLI1 (Ser-102, Ser-408 and Thr-1074) are not located in the β-TrCP degron motif (DSGVEM) [8], which illustrates how multiple phosphorylation codes in a regulatory domain regulate protein-protein interaction. Future studies should assess the regulation of how the phosphorylation sites on GLI1 by AMPK and interact or interfere with those generated by other kinases. Our finding demonstrated that triple phosphorylation of GLI1 by AMPK on Ser-102, Ser-408 and Thr-1074 increases the cytoplasmic localization and binding stability to E3 ligase β-TrCP, ultimately driving GLI1 protein degradation. In particular, understanding the mechanisms balancing GLI1 stability versus degradation may have therapeutic value for cancers in which GLI1 is overexpressed.

## MATERIALS AND METHODS

### Reagents and plasmids

AMPK activator 2DG and inhibitor compound Comp C were purchased from Sigma. CHX and MG-132 were purchased from Sigma. All GLI1 plasmid DNA was generated as previously described [33]. β-TrCP1 WT and Δ mutant DNAs were gifts from Dr. Mien-Chie Hung.

### Cell lines and brain tumor tissue array

NIH3T3 and DAOY cells were obtained from ATCC. pZp53Med1 (Med1) cells were obtained from Dr. Phil Beachy [47]. β-TrCP wild-type and null MEFs were gifts from Dr. Keiko Nakayama [48]. Brain tumor tissue array was purchased from Biomax (Rockville, MD).

### Cell culture, MTT cell growth, and colony formation assay analysis

NIH3T3 was cultured in DMEM supplemented with 10% BCS at 5%CO_2_. All other cell cultures were kept in DMEM supplemented with 10% FBS at 5% CO_2_. The concentrations and time for each chemical treatment were as follows: 2DG (25 mM, 4 hours) and CHX (1 μg/ml), unless otherwise noted. The cell growth rate was determined using MTT and cell counting assays [49]. Cells (3 × 10^3^ per well) were plated in 96-well culture plates. After cells adhered and were maintained for three days, MTT substrate (20 μM) was added and incubated for 2 hours before 100 μl lysis buffer (20% SDS in 50% *N,N*-dimethylformamide at pH 4.7) was added. After the incubation, absorbance was measured at 570 nm. The CellTiter 96® AQueous Non-Radioactive Cell Proliferation Assay Kit was purchased from Promega. For colony formation assays, 5 × 10^4^ cells were placed in 1.5 ml medium and 0.3% agarose and overlaid onto 3 ml medium and 0.6% agarose in each well of a six-well plate and medium was changed every 3 days until the end of the assay. After 2–3 weeks, colonies larger than 2 mm in diameter were counted.

### Immunofluorescence staining and immunohistochemistry staining

Cells were fixed in 4% paraformaldehyde in phosphate-buffered saline (PBS) for 90 min and then permeabilized for 5 min in PBS containing 0.1% Triton X-100. Cells were sequentially incubated with primary antibodies anti-Flag (1:500 dilution) followed by secondary antibodies diluted in bovine serum albumin-containing PBS as a blocking buffer. Cells were mounted in mounting solution. The immunoperoxidase staining method was modified from the avidin-biotin complex technique as described previously [50]. In brief, slides (5 μm) were deparaffinized. After antigen retrieval, the slides were digested in 0.05% trypsin. The endogenous peroxidase activity was blocked by incubation in 0.3% hydrogen peroxide, and the slides were then treated with 10% normal goat or horse serum for 30 min. After overnight incubation with primary antibodies, including (*a*) rabbit polyclonal anti-GLI1 (H-300; 1:100 dilution; Santa Cruz Biotechnology Inc., Santa Cruz, CA); (*b*) rabbit polyclonal anti-phospho-AMPK (T172) (1:50 dilution; Cell Signaling Inc.), the slides were incubated with biotinylated secondary antibodies and subsequently incubated with avidin-biotin-horseradish peroxidase complex (Vector Laboratories, Burlingame, CA). Antibody detection was performed with the 0.125% aminoethylcarbazole chromogen substrate solution (AEC substrate) from Sigma Chemical Co. After counterstaining with Mayer's hematoxylin (Sigma), the sides were mounted. For negative controls, all incubation steps were identical except that PBS instead of primary antibody was used.

### Immunoblotting and immunoprecipitation (IP) assays

Immunoblotting and IP were performed as previously described [49], with the following antibodies: Gli1 and GFP (Santa Cruz Biotechnology); Gli1, AMPK, p-AMPK, ACC, p-ACC, β-TrCP (Cell Signaling); β-Actin, Tubulin, Flag-M2 (Sigma) and HA (Roche). The primary antibody was diluted for 1:1000 in immunoblotting experiments and applied with a final concentration of 1μg for 1mg total protein in immunoprecipitations.

### Lentivirus infection

pCDH-CMV-MCS-EF1-Puro GLI1-lentivirus packaging and infection were performed according to the manual from SBI System Bioscience (Cat. #sCD500–CD700). Infected NIH3T3 cells were treated with 2.5 μg/ml puromycin for two weeks to eliminate non-infected cells.

### Statistical analysis

SPSS and mircosoft excel softwares were used for statistical analysis. A univariate analysis was used to determine the variable distributions. Categorical variables among the groups were compared with the Pearson's chi-square test. A *p*-value of < 0.05 was considered statistically significant.

## SUPPLEMENTARY MATERIALS FIGURES


