# Supplementary material for: Dual degradation signals destruct GLI1: AMPK inhibits GLI1 through β-TrCP-mediated proteasome degradation
Source: Oncotarget. 2017 May 10;8(30):49869–81. doi: 10.18632/oncotarget.17769 (PMC5564814; doi:10.18632/oncotarget.17769)
Supplement: Supplementary file 1 [file oncotarget-08-49869-s001.pdf]

Dual degradation signals destruct GLI1: AMPK inhibits GLI1 through β-TrCP-mediated proteasome degradation

SUPPLEMENTARY MATERIALS

SUPPLEMENTARY FIGURES

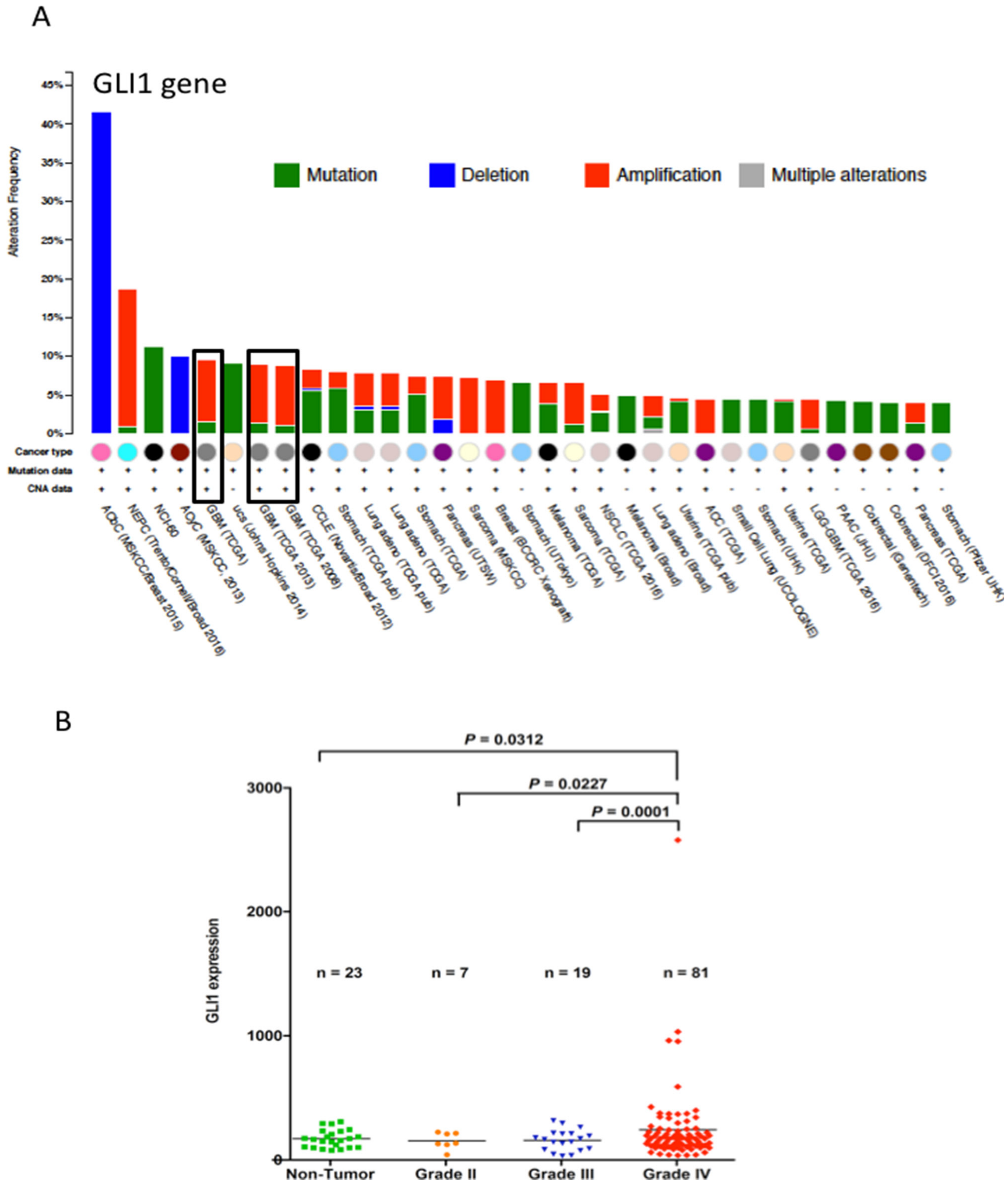

Supplemental Figure 1: GLI1 gene profile and expression in human cancers. (A) A cross-cancer human GLI1 genomic analysis from 147 studies from the Memorial Sloan Kettering Cancer Center database (cBioPortal for Cancer Genomics). **(B)** GLI1 gene expression is analyzed by gene expression omnibus (GEO) from the NCBI website in a group of glioma patients.

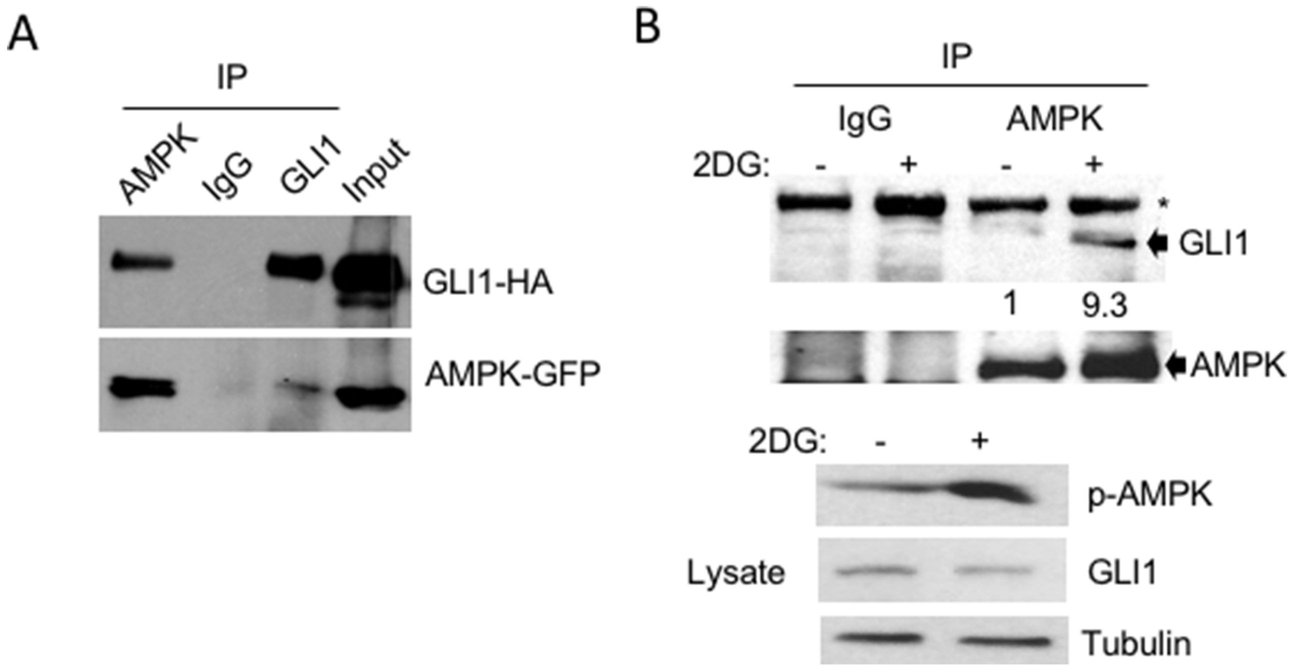

Supplemental Figure 2: Physical association of AMPK and GLI1. (A) HEK293T cells were co-transfected with HA-tagged GLI1 and GFP-tagged AMPK and the lysates were immunoprecipitated with antibody against HA and GFP, and immunoblotted with anti-HA and GFP antibody. **(B)** pZp53Med1 (Med1) cells were treated with 2DG (25mM) for 30 minutes to induce AMPK activity but didn't affect GLI1 stability. Immunoprecipitation (IP) was performed with antibodies against AMPK and Western blots were performed with GLI1 and AMPK antibodies to detect the interaction of GLI1 and AMPK. The numbers, from Image J analysis, indicate the fold increase of GLI1 protein levels in the 2DG treated cells compared to non-treated cells.

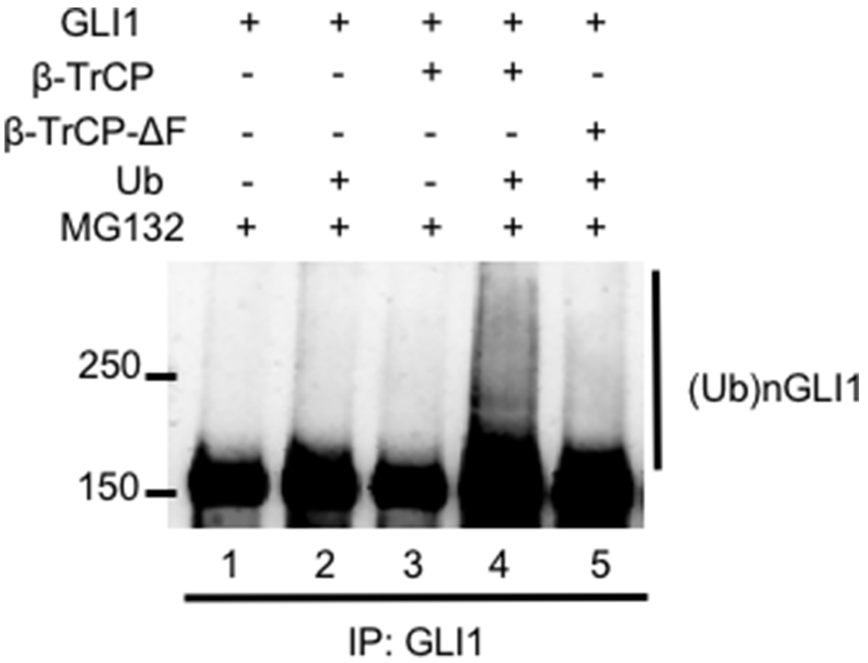

Supplemental Figure 3: β-TrCP ubiquitinates GLI1. Myc-tagged β-TrCP and β-TrCP-ΔF were co-transfected with GLI1 and ubiquitin (Ub) in HEK293 cells for 36 hours and treated with MG132 for 6 hours prior to cell lysis. GLI1 ubiquitination was analyzed by Western blotting.

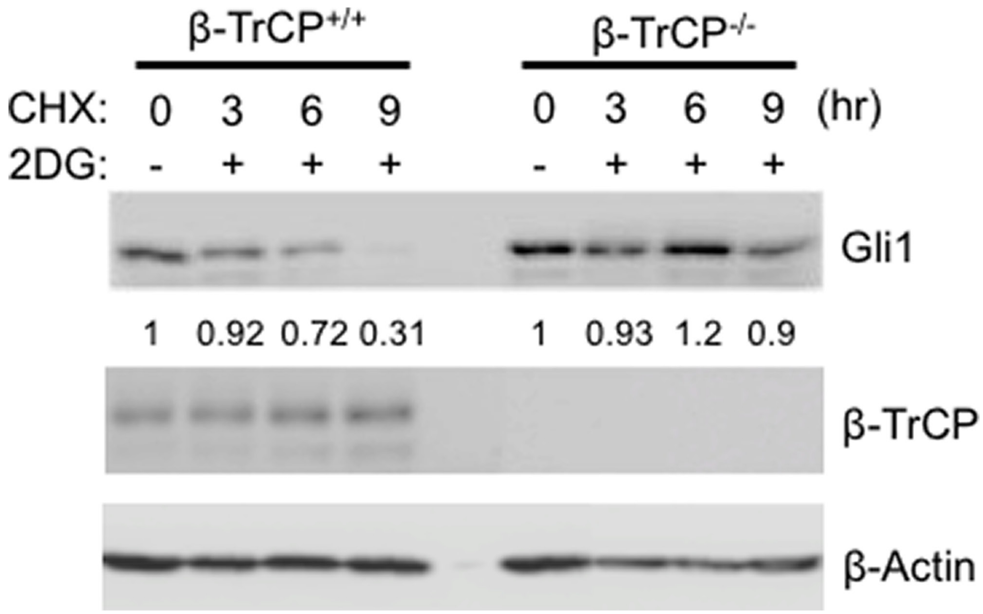

Supplemental Figure 4: AMPK and β-TrCP control GLI1 stability. Lysates of β-TrCP+/+ and β-TrCP-/- MEF cells were harvested at indicated time points after co-treatment with cyclohexamide (CHX, 1 ug ml-1) with or without 2DG (25mM). GLI1 expression was analyzed by immunoblotting. The annotated number indicates the ratio of GLI1 protein over β-actin protein levels as analyzed by Image J.
